# A Molecular Toolkit to Visualize Native Protein Assemblies in the Context of Human Disease

**DOI:** 10.1038/srep14440

**Published:** 2015-09-23

**Authors:** Brian L. Gilmore, Carly E. Winton, Andrew C. Demmert, Justin R. Tanner, Sam Bowman, Vasilea Karageorge, Kaya Patel, Zhi Sheng, Deborah F. Kelly

**Affiliations:** 1 Virginia Tech Carilion Research Institute, 2 Riverside Circle, Roanoke, Virginia 24016, United States; 2 School of Biomedical Engineering and Science, Virginia Tech, Blacksburg, VA 24061, United States; 3 Virginia Tech Carilion School of Medicine, 2 Riverside Circle, Roanoke, Virginia 24016, United States; 4 Department of Biological Sciences, Virginia Tech, Blacksburg, VA 24061, United States

## Abstract

We present a new molecular toolkit to investigate protein assemblies natively formed in the context of human disease. The system employs tunable microchips that can be decorated with switchable adaptor molecules to select for target proteins of interest and analyze them using molecular microscopy. Implementing our new streamlined microchip approach, we could directly visualize BRCA1 gene regulatory complexes from patient-derived cancer cells for the first time.

A current limitation in decoding the exquisite interactions that occur in human cells is the lack of molecular techniques to investigate protein assemblies formed in their native environment. In the context of human disease, this is particularly challenging. Standard separation procedures to acquire protein assemblies are limited, in part, to the lengthy chromatographic steps^1^ that employ harsh chemicals to obtain the fragile biological output. While functionalized surfaces have been successfully used to isolate proteins from model expression systems^2^,^3^, a novel strategy is to utilize Silicon Nitride (SiN) microchips in a versatile manner to recover clinically relevant proteins from human cells in a pathological state. To accomplish this task, we developed a new molecular toolkit using patient-derived cancer cells that produce the breast cancer susceptibility protein, BRCA1. We elected to use this system because *(1)* mutations in the gene encoding for BRCA1 are heavily linked to the development of familial breast and ovarian cancers^4^,^5^, accounting for ~25% of all diagnosed cases^6^,^7^,^8^; *(2)* the manner in which BRCA1 works in concert with other protein machinery is ill-defined; *(3)* molecular structures of complexes containing BRCA1 are heavily under-investigated despite their clinical significance. During normal cellular activities, the BRCA1 protein associates with its binding partner, BARD1 (BRCA1-Associated Ring Domain protein), to assist with genomic repair events and RNA synthesis^9^. Although BRCA1 plays a critical role in these processes, the precise manner in which BRCA1 interacts with other proteins remains unclear.

## Results and Discussion

### BRCA1 associates with the RNAP II core in hereditary breast cancer cells

**Figure 1:**
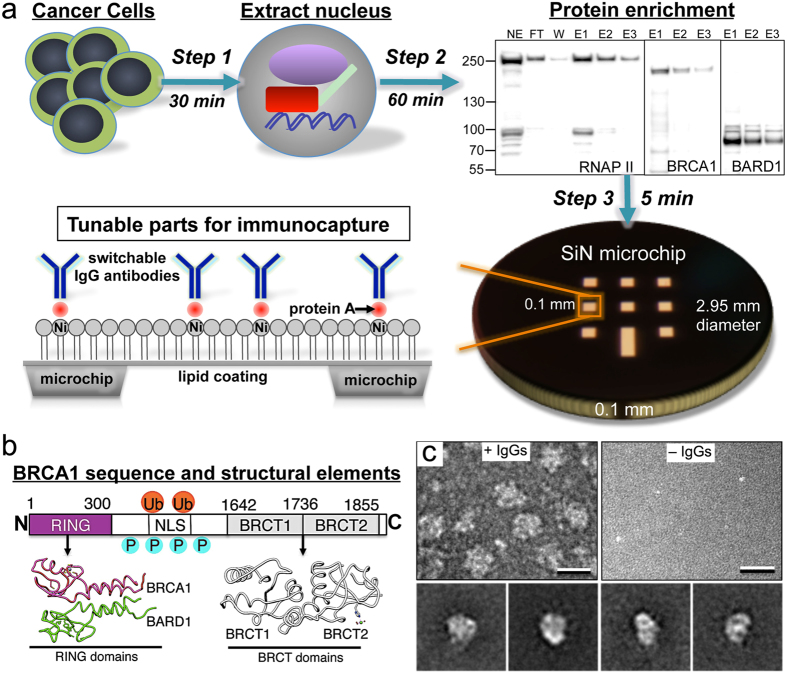
The tunable microchip approach to capture native protein assemblies formed in human cancer cells. (**a**) Breast cancer cells were lysed and the nuclear material was separated from the other cellular contents (**Step 1, 30 min**) and enriched (**Step 2, 60 min**) in RNAP II (RPB1 subunit, ~260 kDa), BRCA1 (~220 kDa) and BARD1 (~87 kDa) as identified by western blot analysis. The enriched material was applied to tunable microchips (**Step 3, 5 min**) coated with a Ni-NTA lipid layer and protein A, along with switchable IgG antibodies. (**b**) The primary sequence of BRCA1 is composed of the N-terminal RING domain (magenta) that forms a functional dimer with BARD1 (green; pdb code, 1JM7) while the central region contains nuclear localization sequences (NLS) and sites for phosphorylation (P) and ubiquitination (Ub). The C-terminus of BRCA1 is composed of two tandem BRCT domains (gray; pdb code, 1JNX). (**c**) EM images and representative class averages (**bottom panel**) of specimens prepared on tunable microchips in the presence **(top, left panel)** and absence **(top, right panel)** of IgG antibodies against BRCA1 demonstrate the specificity of the method. Scale bar is 20 nm. The width of each panel of averages is 36 nm.

To better define BRCA1 interactions in a hereditary breast cancer system, we interrogated the nuclear contents of primary ductal carcinoma cells that express wild type BRCA1 (HCC70 line) (Fig. 1a**, Step 1**). Protein complexes that contain active transcriptional assemblies can be naturally enriched upon binding to immobilized-metal affinity matrices^10^. Consistent with this observation, RNA Polymerase II (RNAP II) – the machinery responsible for mRNA production in all human cells – BRCA1, and BARD1 contained in nuclear extracts co-eluted from Nickel-nitrilotriacetic acid (Ni-NTA) agarose beads (Fig. 1a**, Step 2**).

We tested for protein-protein interactions in the eluted fractions by performing co-immunoprecipitation (co-IP) experiments and western blot analysis. These results showed RNAP II complexes contained in the enriched nuclear fractions can interact with BRCA1 (Supplementary Fig. 1a) and that the large subunit of RNAP II (RPB1) was phosphorylated at serine 5 (pSer5) and serine 2 (pSer2) positions, suggesting the complexes were in an active state^11^. We further detected that K63-linked ubiquitin moieties were present at ~260 kDa and coincident with RPB1 migration on denaturing gels. This result indicated that the phosphorylated RPB1 subunit may also contain K63-ubiquitins moieties, a known signal for DNA repair^12^ (Supplementary Fig. 1b). K48-linked ubiquitin moieties that signal for protein degradation were not detected. We then used the enriched fractions as input for our microchip experiments (Fig. 1a**, Step 3**).

To produce tunable surfaces, we coated naturally hydrophobic SiN microchips having 10-nm thin windows (TEMwindows) with lipid films comprised of Ni-NTA-functionalized phosphatidyl head groups (Avanti Polar Lipids). For negatively stained samples, we used 5% Ni-NTA films and for cryo-EM specimens we used 25% Ni-NTA films while employing 1,2-dilauroyl-*sn*-glycero-3-phosphocholine (DLPC) filler material (Avanti Polar Lipids). Each lipid component was constituted to 1 mg/ml in chloroform. The coated microchips were incubated for 1 minute with aliquots (3-μl each) of protein adaptors including His-tagged protein A (AbCam) and switchable IgG antibodies raised against either the N-terminal RING or BRCT domains of BRCA1 (Fig. 1b). His-tagged Protein A (AbCam) and the antibody adaptors were each diluted to a working concentration of 0.01 mg/ml into buffer solution containing 50 mM HEPES (pH 7.5), 150 mM NaCl, 10 mM MgCl_2_, and 10 mM CaCl_2_. The antibody solutions were added to the Protein-A coated microchips and incubated for 1 minute, after which time the excess solution was gently removed with a Hamilton syringe.

To test the specificity of our tunable microchips, we added aliquots (3-μl) of the enriched nuclear material (fractions E1–E3, ~0.3 mg/ml) to microchips decorated with or without the antibodies against BRCA1. Following a 2-minute incubation step with the microchips, the excess solution was removed and either negatively stained with 0.2% uranyl formate for antibody-labeling analysis or plunge-frozen into liquid ethane using a Cryoplunge 3 device equipped with GentleBlot technology (Gatan, Inc.). We refer to SiN used for cryo-EM as “Cryo-SiN”. Images and class averages of antibody-decorated microchips incubated with the enriched nuclear fractions revealed specific protein complexes were present in sufficient quantities (Fig. 1c**, left and bottom panels**). Protein A-decorated microchips lacking antibodies generally lacked complexes (Fig. 1c**, right panel**). Overall, these results indicated that antibody-decorated microchips could be used to recruit BRCA1-associated complexes from the fractionated nuclear material.

A key advantage of using the microchip capture system is that it required only a 30-minute nuclear extraction, a ~60-minute fractionation of the nuclear material, and a ~5-minute tethering step. This totals ~95 minutes from lysing the cells to preparing the specimens. Thus, our streamlined approach presents a major departure from classical procedures that require days to complete. Another advantage of the system is that transcriptional complexes could be attached to the microchips via the BRCA1-specific antibodies, thus facilitating adequate BRCA1 occupancy in associated structures.

### The first structural information of BRCA1 protein assemblies formed in human cancer cells

**Figure 2:**
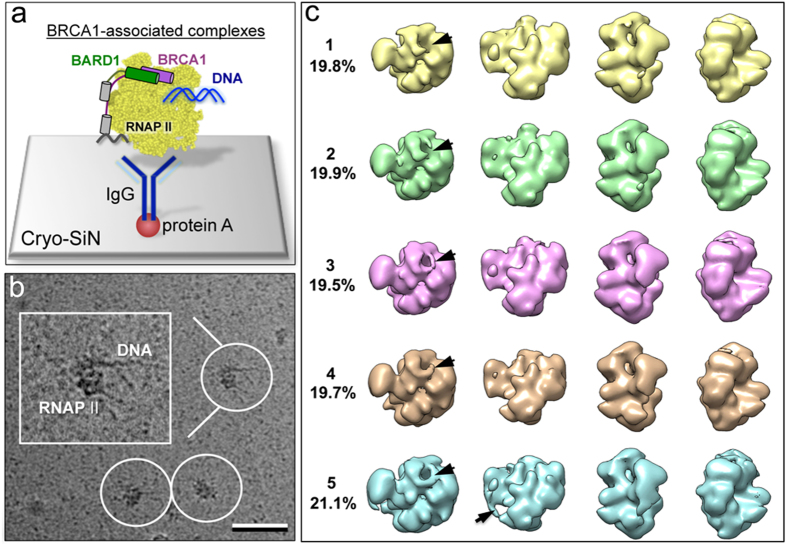
BRCA1-associated transcriptional complexes sorted by 3D classification. (**a**) Schematic illustration of the rapid capture technique used to tether BRCA1-associated complexes to Cryo-SiN microchips. (**b**) Representative cryo-EM image of BRCA1-containing RNAP II complexes bound to DNA. Scale bar is 50 nm. (**c**) 3D classification of EM structures independently identified by the RELION software package^13^. Each structure shows subtle variations in electron density, some of which are indicated by black arrows. The percentage of particle images contained within each structure is indicated.

In order to directly visualize the BRCA1-associated complexes tethered to the Cryo-SiN microchips via the BRCT antibodies, we collected low-dose images (~5 electrons/Å^2^) using a FEI Spirit BioTwin Transmission Electron Microscope (TEM) (FEI Company) equipped with a LaB_6_ filament operating at 120 kV. Using automated routines in the PARTICLE software package (http://www.image-analysis.net/EM/), we selected from the images ~22,000 individual BRCA1-transcriptional complexes that were bound to DNA (Fig. 2a,b). The selection criteria implemented in PARTICLE did not readily identify proteins consistent with the size of BRCA1 alone or BRCA1-BARD1, as these entities were small in comparison to the full BRCA1-associated RNAP II core complexes. The selected BRCA1-transcriptional assemblies were exported as an image stack into the RELION software package^13^ that was used to refine and reconstruct the selected complexes while employing an initial model for RNAP II^14^ (pdbcode, 4A93).

**Figure 3:**
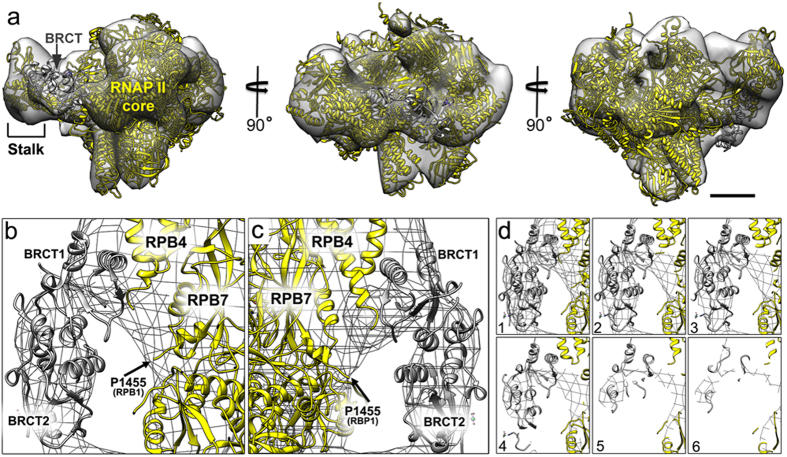
The composite EM reconstruction with the BRCT domain positioned in the density map. (**a**) The EM density map is shown in different orientations with the RNAP II core (yellow; pdbcode, 4A93^3^) and BRCT domain (gray; pdbcode, 1JNX^6^) positioned in the map. The RNAP II stalk domain that consists of subunits RBP4 and RPB7 is also shown within the density map. Scale bar is ~10 nm. (**b**) A close-up view of the BRCT domain (gray; pdbcode, 1JNX^6^) composed of BRCT1 and BRCT2 motifs displayed in two opposing views (**b,c**) with respect to the RNAP II subunits (yellow; pdbcode, 4A93^3^) that define the stalk domain (RPB4/RPB7). The RNAP II large subunit (RPB1) and its terminal residue defined in the crystal structure (P1455; black arrows) are also indicated. **(d)** Sections (1–6) through the BRCT domain positioned within the EM density map. Please also see Supplementary Movie 1.

We performed 3D classification routines to assess the degree of intermediates present in our image stack. We used the RELION software package to independently identify variable 3D structures. Based on statistical likelihood comparisons computed between the particle images and that of the initial model, five distinct structures were output, independent of the user-defined starting parameters. The five 3D structures varied slightly in a few regions of density, some of which are indicated in Fig. 2c by black arrows. Overall, the cryo-EM specimens did not show strongly preferred orientations as verified in the angular distribution plots of particle projections that comprised each reconstruction. These plots were generated in RELION (.BILD format) during the final cycle of refinement (Supplementary Fig. 2). Color designations in the plots from blue to red indicate an increase in the number of particles at a particular coordinate. This information enabled us to calculate a composite 3D structure by combining all of the particles. The resolution of the final structure was ~2.2 nm using the gold standard FSC criteria (0.5) in RELION (Supplementary Fig. 3a) and was masked at ~250 Å (Fig. 3a, Supplementary Fig. 3b).

To determine the relative locations of the BRCA1 N- and C-terminal domains within the density map, we employed additional computational procedures^15^. For this analysis, we used the SPIDER software package^16^ to select and calculate averages of negatively stained protein complexes labeled with antibodies against either the BRCA1 RING or the BRCT domains. The antibody-labeled complexes revealed additional densities in comparison to computed 2D projections of the RNAP II model that lacked BRCA1-BARD1. The additional densities were attributed to the BRCA1-BARD1 RING domains or the BRCT domain (Supplementary Fig. 3c,d), depending upon the antibodies used to prepare the specimens. We confirmed two unoccupied densities were also uniquely present in the EM density map and were not present in the RNAP II structure that lacked BRCA1. We attributed each of the additional densities in the 3D map (Supplementary Fig. 3b) to the presence of the ordered RING or BRCT domains.

We assigned the unoccupied density proximal to the C-terminus of the RPB1 subunit to the BRCT domain^17^ (Fig. 3a–d, Supplementary Figure 4, Movie 1; gray; pdbcode, 1JNX). This interpretation was based on the antibody-labeling results (Supplementary Fig. 3) and the fact that the BRCT domain is known to associate with this region of RPB1^18^. This new information for the precise position of the BRCT domain with respect to the RPB1 subunit is significant as many cancer-related mutations in the *BRCA1* gene occur within the BRCT domain. Improving our understanding of these mutations at the molecular level may provide an essential framework for future therapeutic development.

### How does the *BRCA1* ^*5382insC*^ clinical mutation affect interactions with protein machinery?

**Figure 4:**
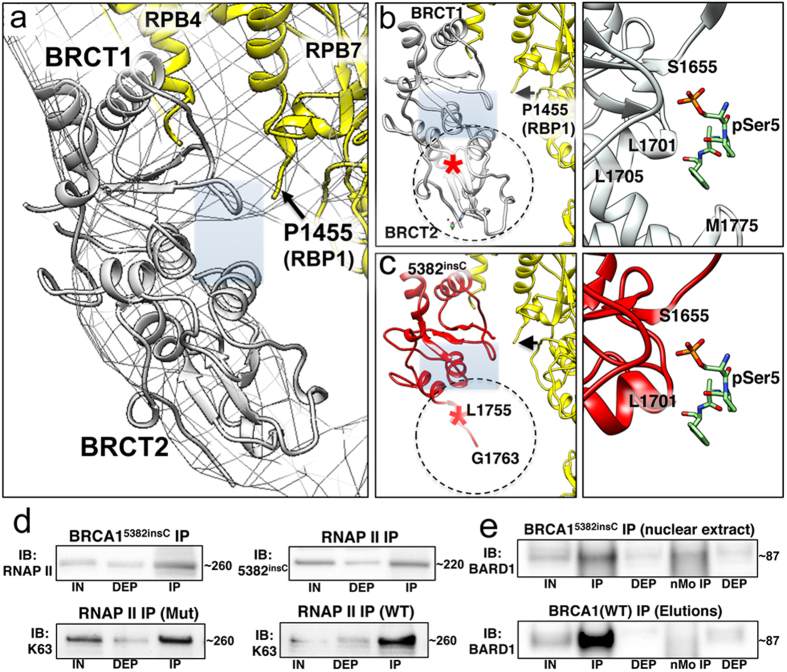
The *BRCA1*^*5382insC*^ mutation likely alters interactions with RNAP II and BARD1. (**a**) Close-up view of the BRCT density with respect to the RPB1 subunit of RNAP II that is disordered beyond P1455 (black arrow), where the C-terminus emanates. Theoretical molecular models of the BRCT domain for wild type BRCA1 (gray; pdbcode, 1KOH) (**b**) compared to the mutated BRCA1^5382insC^ (red) (**c**) revealed the hydrophobic binding pocket (gray rectangle) is disrupted in the mutated BRCT domain. This significant disruption in the peptide-binding site suggests that native substrates may not interact with BRCA1^5382insC^ in the same manner as with wild type BRCA1. (**d**) Western blot analysis indicates that RNAP II (RPB1 subunit) interacts with BRCA1^5382insC^ in co-IP experiments, and the RNAP II core is similarly ubiquitinated by K63-specific moieties in cell lines expressing both mutated (Mut) and wild type (WT) BRCA1. The large subunit of the RNAP II core (RPB1) migrates at ~260 kDa. BRCA1 migrates at ~220 kDa. (**e**) The BRCA1^5382insC^ protein contained in nuclear extracts showed some interaction with BARD1 in comparison to negative control IPs performed using species-specific mouse normal (nMo) IgG antibodies (top panel). Wild type (WT) BRCA1 shows a strong interaction with BARD1 in Ni-NTA eluted fractions used as input material for microchip-capture experiments (bottom panel). BARD1 migrates at ~87 kDa. IN (input material); DEP (unbound); IP (immunoprecipated protein); IB (immunoblot).

To better define how the prevalent *BRCA1*^*5382insC*^ clinical mutation affected interactions with other proteins, we compared the structure of the wild type BRCT^17^ (Fig. 4a,b) to a homology-based model of the mutated BRCT (Fig. 4c). In the homology model, S1755 was mutated to L1755 (Fig. 4c, red star; Supplementary Fig. 5a) due to the frame-shift in the DNA sequence. We could generate a polypeptide structure up to residue G1763, although we found no secondary structure beyond G1763. Upon examining the phosphopeptide binding site in the mutated BRCT model, we noted the essential hydrophobic pocket was significantly disrupted in comparison to the wild type peptide-binding site (Fig. 4b, Supplementary Movie 2). Based on these insights, the *BRCA1*^*5382insC*^ mutation likely alters interactions with proteins that bind to this region including RNAP II (Fig. 4c, Supplementary Movie 3).

To test this theoretical model experimentally, we utilized primary ductal carcinoma cells (HCC1937 line) that express a homozygous form of the mutated protein, *BRCA1*^*5382insC*^, and that have limited transcriptional repair activity^9^. We assessed biochemical interactions among protein assemblies containing mutated BRCA1 in comparison to wild type BRCA1, using the same amount of nuclear material in each analysis. For the complexes derived from HCC1937 cells, we found that RNAP II and BRCA1^5382insC^ were each present in the enriched nuclear fractions in similar quantities to wild type proteins (HCC70 cells). However, we noted a decline in BARD1 levels in the enriched nuclear fractions of cells that express BRCA1^5382insC^, compared to cells that express wild type BRCA1 (Supplementary Fig. 5b). Co-IP experiments on the corresponding fractions revealed that despite the frame-shift mutation in the BRCT domain, BRCA1^5382insC^ still associated to some extent with phosphorylated RNAP II complexes containing K63-ubiquitn moieties (Fig. 4d). These results support the idea that the N-terminal RING domain of BRCA1 must play an important role in maintaining interactions with the RNAP II core^19^.

Next, we noted that decreased levels of BARD1 in the Ni-NTA eluted fractions made it difficult to assess its interactions with other proteins, suggesting that BARD1 associated less efficiently with BRCA1^5382insC^. BARD1 did associate with BRCA1^5382insC^ in the complete nucleus extract, albeit at reduced levels compared with wild type BRCA1 affiliated with transcriptional complexes in the enriched nuclear fractions (Fig. 4e). Therefore, this data is in good agreement with previous reports specifying that BARD1-BRCA1 dimerization requires a functional BRCT domain, and that mutations in the BRCT may disrupt these critical associations^20^. Collectively, these results can serve as the basis for further developing tunable microchips to assess protein complexes formed in patient-derived tumor samples harboring a variety of *BRCA1* mutations.

Overall, here we demonstrate that the use of tunable microchips provides a powerful new means to directly assess native protein assemblies relevant to human cancer. The robust nature of this approach has also been recently demonstrated at the cellular level^21^. By decorating SiN microchips with antibodies against the NOTCH1 protein receptor that is overexpressed on the surface of glioblastoma stem cells (GSCs), we could isolate GSCs from a heterogeneous population of primary brain tumor cells. This cellular isolation/tethering step enabled us to record the first real-time movies of GSCs interacting with gold nanoparticles at the molecular level using *in situ* TEM^21^.

Based on the fact that our new tools can be used to analyze cellular and molecular aspects of patient-derived tumor samples from different diseased conditions, we anticipate these tools can be easily adapted to study other pathologies. Such conditions may include but are not limited to neurodegeneration, cardiac myopathies, immune response deficiencies, and host-pathogen interactions. Future efforts to unravel disease-related protein interactions may also lead to new opportunities for therapeutic targeting in a manner that has not been fully realized. Ultimately, when used in combination with other bioinformatics tools, the tunable microchip approach may shed light on disease processes in a unique manner that is currently lacking in traditional methods of scientific and clinical inquiry.

## Methods

### The preparation of BRCA1-associated complexes from human tumor cells

HCC70 and HCC1937 cells (ATCC) were grown to near confluence in RPMI-1640 medium (Mediatech) supplemented with 10% fetal bovine serum (Fisher Scientific) in a 5% CO_2_ atmosphere at 37 **°**C. Cells were detached with trypsin-EDTA (Life Technologies) followed by a brief centrifugation (500 *xg*, 5 min.), washed with PBS and pelleted. The NE-PER extraction kit (Thermo Scientific) was used to separate cytoplasmic and nuclear fractions. Nuclear extract (NE) was diluted in HEPES buffer (20 mM HEPES, 2 mM MgCl_2_, 2 mM CaCl_2_, pH 7.2) to approximately 1 mg/ml supplemented with 5 mM imidazole and protease inhibitor cocktail (EDTA-free, Roche). Diluted NE (~1 mg/ml) was added to pre-equilibrated Nickel-nitroltriacetic acid (Ni-NTA) agarose beads (Qiagen) and incubated on a rotator for 60 minutes at 4 ^o^C. The mixture was pooled into a column and the flow-through was collected for analysis. The column was washed three times with HEPES buffer supplemented with 140 mM NaCl and 5 mM imidazole. Proteins were eluted with HEPES buffer with NaCl supplemented with 150 mM imidazole. Protein concentration was estimated using the Bradford assay (Thermo Scientific).

### Co-IP experiments

Ni-NTA eluates were pooled to obtain 200 μg of total protein per immunoprecipitation and supplemented with protease inhibitor and phosphatase inhibitor cocktail (Thermo Scientific). Five micrograms of antibody diluted in PBS-T (0.02% Tween-20, Fisher) was added to 0.75 mg Dynabeads Protein G (Life Technologies). For co-IP analysis, we used antibodies raised against either the RPB3 subunit of RNAP II (AbCam, ab83098), or the BRCA1 C-terminal (BRCT) region (Santa Cruz Biotechnology, C-20), and normal mouse IgG (SCBT sc-2025) as immunoprecipitations. The mixture was incubated with rotation for 30 minutes at 4 °C. The antibody-coated beads were subsequently washed in HEPES buffer prior to adding pooled eluates. Protein was immunoprecipitated overnight at 4 °C with gentle rotation. The beads were then washed three times with HEPES buffer followed by elution with NuPAGE LDS sample buffer. Proteins were separated on 4–12% NuPAGE Bis-Tris mini gels with MOPS running buffer before transferring onto an Immobilon-P membrane (Millipore) in a Mini-PROTEAN Tetra system (Bio-Rad). Blots were blocked with a 1% non-fat dry milk (NFDM) or 4% bovine serum albumin (BSA, SCBT) solution for 1 hour with gentle rocking. Primary antibody was diluted in 1% NFDM or BSA solution and incubated overnight at 4^o^C. Additional antibodies employed were RNAP II (SCBT sc-9001, H-224), RNA Polymerase II H5 (pSer2-specific) and H14 (pSer5-specific) (Covance MMS-129 and MMS-134), BRCA1 N-terminal (RING) domain (Millipore, AB1, MS110), Polyubiquitin (K63-linkage-specific, Enzo BML-PW0600) and ubiquitin (K48-linkage-specific, AbCam ab140601). Blots were washed three times with TBS-T (0.05%). Goat anti-rabbit or goat anti-mouse secondary antibodies conjugated to horseradish peroxidase (Jackson ImmunoResearch) were incubated for 1 hour followed by additional washing with TBS-T. ECL Prime western blotting reagent (GE Healthcare) was used for detection and a ChemiDoc MP (Bio-Rad) for imaging.

### Electron microscopy

We collected images of the BRCA1-associated complexes using a FEI Spirit BioTwin Transmission Electron Microscope (FEI Company) equipped with a LaB_6_ filament operating at 120 kV under low-dose conditions (~5 electrons/Å^2^) for negatively stained and frozen-hydrated samples. We recorded images on a FEI Eagle 2k HS CCD camera (FEI Company) with a pixel size of 30-μm at a nominal magnification of 50,000×, for a final sampling of ~6-Å per pixel. Images of negatively stained and ice-imbedded BRCA1-RNAP II specimens were acquired using the same TEM and under the same conditions, but varying the defocus. For 3D reconstruction calculations using the RELION software package^13^, the initial model was low-pass filtered to 80-Å resolution and was used only in the first phase of the reconstruction routine to assign initial orientation parameters to each particle in the image stack. The following iterations relied heavily on the experimental data with a regularization parameter of *T = 4* in order to refine the assigned angles. We employed a pixel size of 6-Å and a box size of 60 pixels while following standard reconstruction procedures^13^. For negatively stained antibody-labeled specimens, we used the SPIDER software package to perform standard multi-reference alignment routines followed by principle component analysis and K-means classification^16^. To interpret the results, we used the cross-correlation function implemented in the SPIDER software package to match representative averages with filtered 2D projections of the yeast RNAP II crystal structure^14^ (pdbcode, 4A93). We calculated comparisons between the averages and 2D projections of the crystal structure with normalized cross-correlation values greater than 0.8 (scale of 0–1.0; 0 meaning no similarities, 1.0 meaning identical) being considered in similar orientations^15^. The composite EM structure is being made available for download from the EMdatabank (accession code, EMD-6340).

### Homology modeling

The primary sequence of the BRCA1^5382insC^ protein was submitted for homology modeling to the SWISS-MODEL website (http://swissmodel.expasy.org), which output the 3D coordinates of the structure in pdb format.

## Additional Information

**How to cite this article**: Gilmore, B. L. *et al.* A Molecular Toolkit to Visualize Native Protein Assemblies in the Context of Human Disease. *Sci. Rep.* **5**, 14440; doi: 10.1038/srep14440 (2015).

## Supplementary Material

Supplementary Information

Supplementary Movie 1

Supplementary Movie 2

Supplementary Movie 3
