# Supplementary material for: A Molecular Toolkit to Visualize Native Protein Assemblies in the Context of Human Disease
Source: Sci Rep. 2015 Sep 23;5:14440. doi: 10.1038/srep14440 (PMC4585766; doi:10.1038/srep14440)
Supplement: Supplementary Information [file srep14440-s1.pdf]

A Molecular Toolkit to Visualize Native Protein Assemblies in the Context of Human Disease

Brian L. Gilmore[1], Carly E. Winton[1,2], Andrew C. Demmert[1], Justin R. Tanner[1], Sam Bowman[1], Vasilea Karageorge[1], Kaya Patel[1], Zhi Sheng[1], and Deborah F. Kelly[1,2,3]*

[1]Virginia Tech Carilion School of Medicine and Research Institute, 2 Riverside Circle, Roanoke, Virginia 24016, United States

[2]School of Biomedical Engineering and Science, Virginia Tech, Blacksburg, VA 24061, United States

[3]Department of Biological Sciences, Virginia Tech, Blacksburg, VA 24061, United States

Corresponding Author

*Email: debkelly@vt.edu

Supplementary Information

Figures

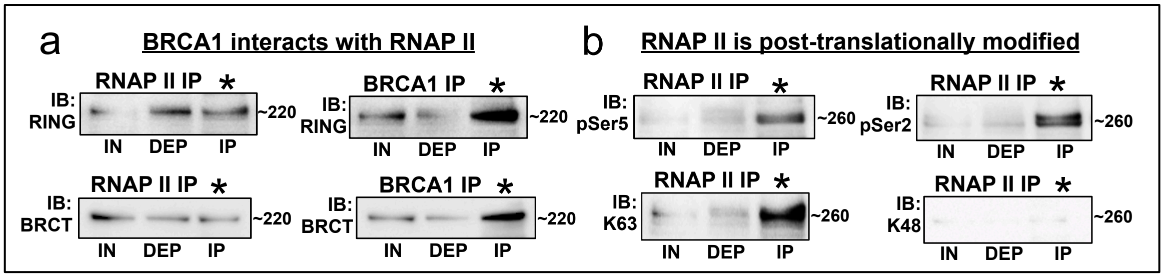

Supplementary Fig. 1. BRCA1 associates with RNAP II in triple negative breast cancer cells. (a) Western blot analysis of co-immunoprecipitation (Co-IP) experiments performed on the enriched nuclear fractions indicated RNAP II complexes interacted with BRCA1, probing with antibodies against the RING and BRCT domains of BRCA1. **(b)** RNAP II complexes in the eluted fractions were post-translationally phosphorylated at pSer5 and pSer2 positions and contained K63-linked ubiquitin moieties. K48-linked ubiquitin was not detected in RNAP II co-IPs. IN (input material); DEP (unbound); IP (immunoprecipitated protein); IB (immunoblot). (*) denotes co-IP results.

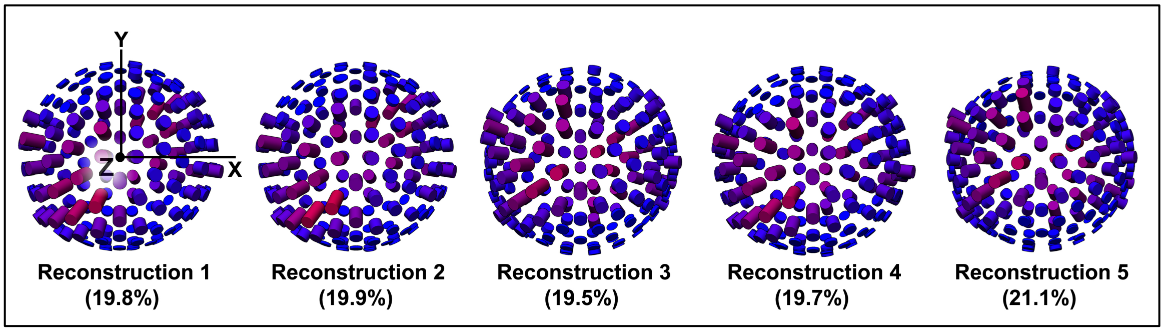

Supplementary Fig. 2. Angular distribution plots of particle projections. RELION output files (.BILD format) specified particle orientations that comprised each reconstruction shown in **Figure 2c**. Color designations from blue to red indicate an increase in the number of particles represented at a particular 3D coordinate. Plots were displayed using the Chimera software package.[1]

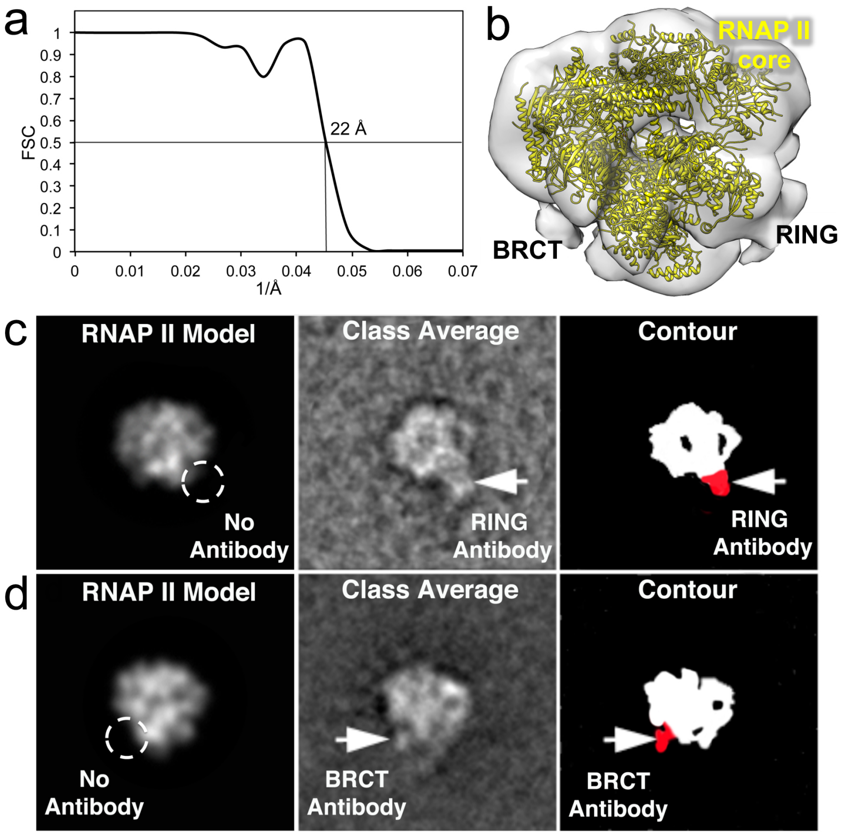

Supplementary Fig. 3. The composite EM structure contains densities that can be delineated using antibody-labeling strategies. (a) FSC curve for the composite EM structure indicates a nominal resolution of ~22 Å using the 0.5 criteria. **(b)** The RNAP II core structure[2] (yellow) was placed uniquely within the EM envelope that contained additional densities that were identified to be the BRCA1-BARD1 RING domains and the BRCT domain. Representative averages of complexes labeled with antibodies against the RING **(c)** or BRCT **(d)** domains of BRCA1 in comparison to a model of the RNAP II crystal structure[2] lacking BRCA1 and associated antibodies. White arrows denote additional density seen in the antibody-labeled averages. Red labeling is used to assist in visually identifying antibody density in contoured representations of the averages. The width of each panel in (c) and (d) is 36 nm.

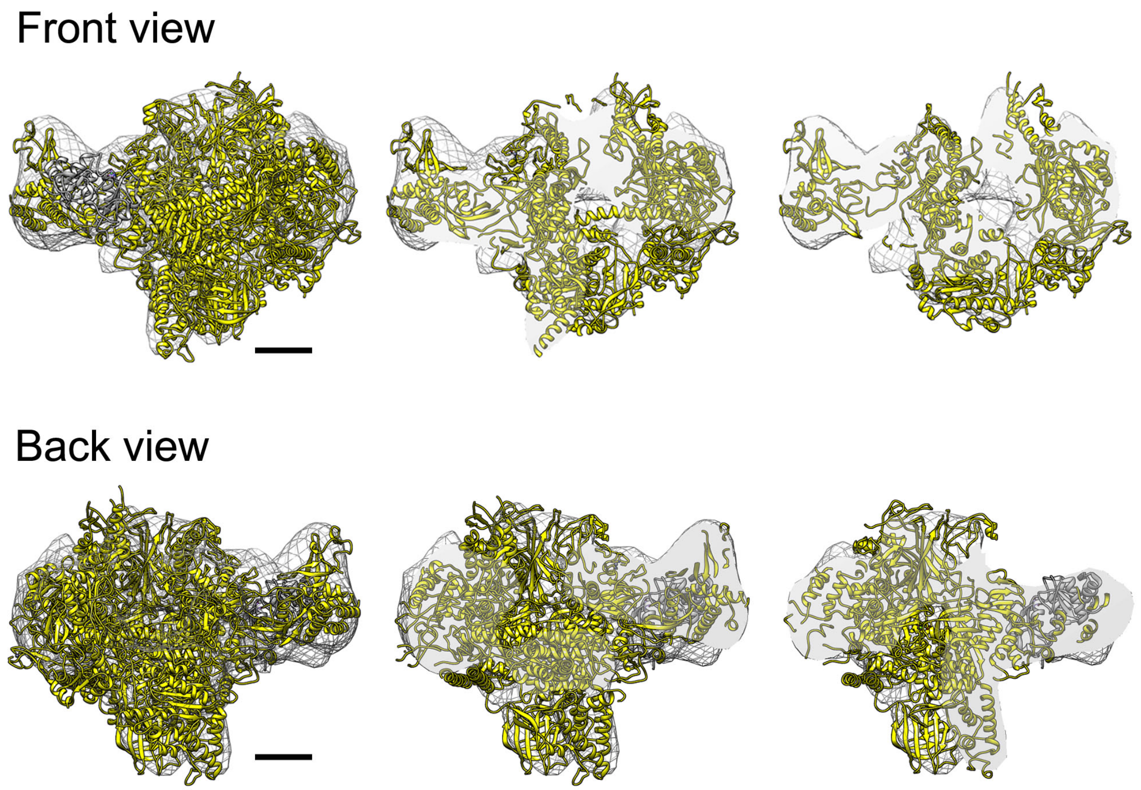

Supplementary Fig. 4. Cross-sections through the composite EM reconstruction. Representative slices through the composite EM density map shown in different orientations (front and back views) with the RNAP II core (yellow; pdbcode, 4A93[2]) and BRCT domain (gray; pdbcode, 1JNX[3]) positioned in the map. Scale bar is 5 nm.

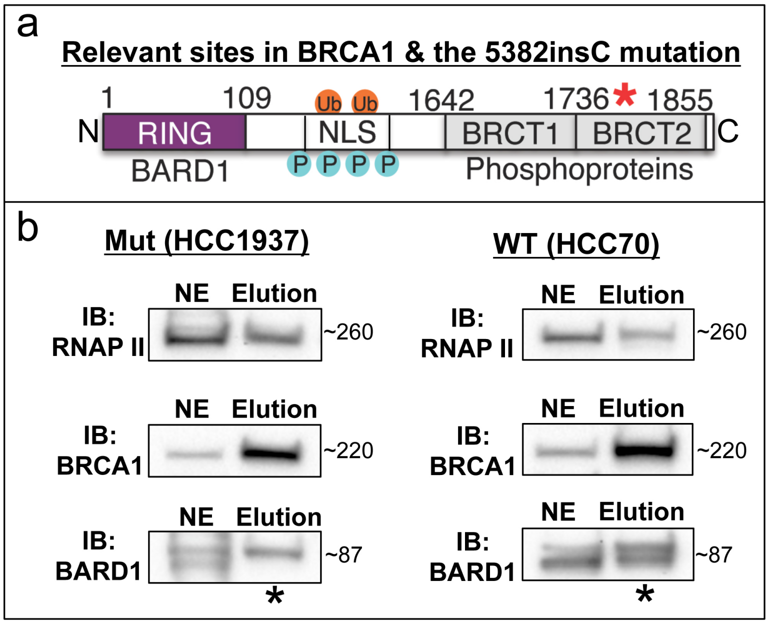

Supplementary Fig. 5. Enriched nuclear fractions containing the *BRCA1*^{5382insC} mutation show differences in BARD1 levels. (a) The primary sequence of the BRCA1 protein indicates the relative location of the *BRCA1*^{5382insC} frame-shift mutation (red star) that occurs in the BRCT domain. **(b)** Western blot analysis indicates that RNAP II (RPB1 subunit) and BRCA1 were present in nuclear extracts (NE) and in Ni-NTA elutions from HCC1937 cells that express the *BRCA1*^{5382insC} mutation (Mut). RNAP II and BRCA1^{5382insC} were present in similar quantities to wild type (WT) proteins produced in HCC70 cells. BARD1 was not as abundant in the enriched fractions containing mutated BRCA1 (HCC1937 cells) compared to wild type BRCA1 (HCC70 cells). (*) denotes differing amounts of BARD1 in the eluted fractions.

Movies

Supplementary Movie 1. Sections through the BRCT domain positioned within the EM density map. The BRCT domain (gray; pdbcode, 1JNX[3]) is displayed with respect to the RNAP II subunits (yellow; pdbcode, 4A93[2]) that define the stalk domain and RPB1, the largest subunit of the complex. This movie accompanies **Fig. 3**.

Supplementary Movie 2. Theoretical model of a phosphopeptide substrate fit within the wild type BRCT binding pocket. The tetrapeptide pSer5 (pSPSY) (pink; pdbcode, 4H3K[4]) model for the C-terminus of RNAP II was computationally overlaid with respect to the pSPTF peptide (cyan). The pSPTF peptide was previously co-crystalized with the BRCT domain (gray; pdbcode, 3KOH[5]). We used the morph conformation function in the Chimera software package[5] to perform the overlay routine.

Supplementary Movie 3. Theoretical model of a phosphopeptide substrate fit within the mutated BRCT^{5382insC} binding pocket. The tetrapeptide pSer5 (pSPSY) (pink; pdbcode, 4H3K[4]) model for the C-terminus of RNAP II was computationally overlaid with respect to the pSPTF peptide (cyan), positioned in the BRCT^{5382insC} homology model (red). We used the morph conformation function in the Chimera software package[1] to perform the overlay routine. Essential hydrophobic contacts were not maintained between the model peptide substrate and the mutated BRCT^{5382insC} binding pocket.

The composite EM structure is being made available for download from the EMdatabank (accession code, EMD-6340).

Supplementary References

1. Pettersen, E. F. *et al*. UCSF Chimera--a visualization system for exploratory research and analysis. *J. Comput. Chem.* **25**,1605-1612 (2004).

2. Walmacq, C. *et al*. Mechanism of translesion transcription by RNA polymerase II and its role in cellular resistance to DNA damage. *Mol. Cell.* **46**, 18-29 (2012).

3. Williams, R. S., Green, R., & Glover, J. N. Crystal structure of the BRCT repeat region from the breast cancer-associated protein BRCA1. *Nat. Struct. Biol.* **8**, 838-842 (2001).

4. Xiang, K., Manley, J. L., & Tong, L. An unexpected binding mode for a Pol II CTD peptide phosphorylated at Ser7 in the active site of the CTD phosphatase Ssu72. *Genes Dev.* **26**, 2265-2270 (2012).

5. Campbell S.J., Edwards, R.A. & Glover, J.N. Comparison of the structures and peptide binding specificities of the BRCT domains of MDC1 and BRCA1. *Structure* **18**, 167-176 (2010).
